# Workplace Hazards Faced by Nursing Assistants in the United States: A Focused Literature Review

**DOI:** 10.3390/ijerph14050544

**Published:** 2017-05-19

**Authors:** AnnMarie Lee Walton, Bonnie Rogers

**Affiliations:** 1 The University of North Carolina at Chapel Hill School of Nursing, 4008 Carrington Hall, CB# 7460, Chapel Hill, NC 27599, USA; 2 North Carolina Occupational Safety and Health Education and Research Center, The University of North Carolina at Chapel Hill Gillings School of Global Public Health, 1700 Airport Road Rm 343, CB# 7502, Chapel Hill, NC 27599, USA; rogersb@email.unc.edu

**Keywords:** nursing assistants, workplace hazards, occupational health

## Abstract

Nursing assistants (NAs) make up a large share of the healthcare provider workforce and their numbers are expected to grow. NAs are predominantly women who earn a low wage and report financial, work, and family demands. Working as a NA is hazardous; this manuscript specifically examines the biological/infectious, chemical, enviromechanical, physical and psychosocial hazards that appear in the literature to date. A focused search strategy was used to review literature about hazards that fell into each of the five aforementioned domains. While some hazards that were documented were clear, such as exposure to influenza because of close contact with patients (biological/infectious), or exposure to hazardous drugs (chemical), literature was limited. The majority of the literature we reviewed fell into the domain of psychosocial hazards and centered on stress from workplace organization issues (such as mandatory overtime, lack of managerial support, and feeling rushed). More research is needed to understand which hazards NAs identify as most concerning and tailored interventions are needed for risk mitigation.

## 1. Introduction

Nursing assistants (NAs) make up a large share of the healthcare provider workforce, and their numbers are expected to grow. NAs are predominantly women who earn a low wage and report financial, work, and family demands. Working as a NA is hazardous; five areas of workplace hazards for this large workforce are explored and described below. The aim of this review is to describe the hazards that appear in the literature in each of these five focused areas. The authors have undertaken this work in order to better understand the nature of these hazards and to develop training strategies and interventions for this target population.

### 1.1. Nursing Assistants

It is estimated that 1,420,570 people work as NAs in the United States (relative standard error 0.6%) [1]. An NA provides basic patient care under direction of a licensed health care provider. In fact, it is estimated that NAs provide 80–90% of direct care to nursing home residents [2]. In nursing homes, as in other settings, a NA performs duties such as feeding, bathing, toileting, dressing, grooming, moving, transferring, turning or repositioning patients, and changing linens. He or she may take vital signs and report clinical changes or concerns of patients to nurses. According to the Bureau of Labor Statistics (BLS), some NAs may dispense medication depending on their training level and state regulations [3].

A focused review of the literature is provided that includes both certified and non-certified nursing assistants. To simplify the language, nursing assistants (NAs) will be used throughout the paper to describe both groups. However, to become a certified nursing assistant (CNA), completion of a certificate program which includes both clinical training and coursework is required and usually occurs after a high school diploma or high school equivalent. These CNA programs can be found in a variety of online schools, community colleges, and trade schools. A competency examination must be passed to earn the CNA designation in addition to registration with the State Board of Nursing. Federal regulations mandate CNAs receive at least 75 h of training and 16 h of clinical training [4]. These regulations apply to those CNAs who are employed by Medicare and Medicaid-certified nursing homes, and over half of all states have chosen to require more than the minimum federal standard training [5]. In some states it is possible to train to be a CNA II, a designation that requires additional training to carry out additional tasks such as sterile dressing changes, tracheostomy care, suctioning, wound irrigation, and gastrostomy feedings [6]. To work in nursing homes and hospitals, certification is required but other titles for passing a state test, being state approved, and being listed on the state registry exist (Registered Nursing Assistant (RNA), Licensed Nursing Assistant (LNA) or State Tested and Approved Nursing Assistant (STNA)). There are some settings in which on-the-job training is provided and registration with the State Board of Nursing is not required, though these jobs are harder to find and may limit an NAs ability to move to another setting afterward.

The majority of NAs are employed in skilled nursing facilities, followed by hospitals, retirement communities, and home health agencies, and a few are employed in alternate industries (scientific research and insurance companies) [1]. Employment of NAs is projected to grow 18% between 2014 and 2024, much faster than the average for all occupations. The aging of the baby-boomer population and the need to care for those people as they age is believed to be the major driver of the growth. The combination of shifts in federal funding for Medicare and Medicaid with patients preferences may lead to a shift from the majority of NAs working in nursing homes to working in community health and rehabilitation services [3].

### 1.2. Demographic Characteristics of Nursing Assistants

Demographic data aside from average income are not available through the Bureau of Labor Statistics. However, a national survey of nursing homes (where the majority of NAs are employed) was conducted last in 2004. The survey, the National Nursing Assistants Survey (NNAS) provides the majority of the demographic data known about NAs. The NNAS was conducted by the long-term care statistics branch of the National Center for Health Statistics [7]. The NNAS was administered to staff at a nationally representative subsample of facilities that participated in the 2004 National Nursing Home Survey (NNHS). Of facilities invited, 81% participated. Among 4542 NAs eligible for inclusion, 3017 completed the survey (71% response rate) [8].

The vast majority of NAs participating in the NNAS were women (92%). While 17% of the respondents were less than 25 years of age and 12% were over age 55, the majority (71%) was fairly evenly distributed between ages 25–34, 35–44 and 45–54. Of NAs participating in the NNAS, 53% were non-white and 47% were white [7]. Nine percent of respondents identified as being of Hispanic or Latino origin. For education data, 44% of the NAs participating had a high school diploma, 19% had 1–3 years of college or trade school, 18% had a high school equivalent certificate, 12% had no high school diploma or high school equivalent, and 5% were graduates of college or had some post-graduate education. There were missing data on education for 2% of the sample.

Nursing assistants earn an average of $27,370 annually. Their mean hourly wage is $12.89 [1]. There is a wide range of earning capacity, with those in the Federal Executive Branch earning an average of $37,450/annually and a NAs working in a nursing facility earning an average of $26,590/annually [1].

In an analysis of NNAS data, it was found that more than one in three NAs received some form of public assistance [9]. Although the median hourly wage is above the federal minimum wage, more than half of NAs’ families are within the 200% poverty level [9]. Direct care workers report worrying about family and especially finances while at work [10].

## 2. Methods

We used a focused search strategy including original research publications and select student dissertations. We used the PubMed journal database and restricted our search to publications in the English language. Due to differences in the workplace organization of nursing homes and hospitals in the United States and elsewhere, for literature that focused on hazards in the workplace we included worksites in the United States only. We did not restrict based on year of publication or methodology; we included quantitative, qualitative and mixed-methods studies. We did not employ systematic data extraction or perform a quality evaluation on the studies we included. Our search terms included (but were not limited to) “nursing assistants and occupational hazards”, “nursing assistants and workplace hazards”, “nursing aides and occupational hazards”, and “nursing aides and workplace hazards”, as well as more focused terms within the five hazards of interest such as “nursing assistants and physical hazards”. Abstracts were reviewed for relevance, and relevant manuscripts were reviewed in full. Relevant articles were also reviewed from the reference lists of collected studies and by those suggested as similar by database searching. Grey literature from institutes and governmental agencies was also used. Examples of some of the agencies are the National Institute for Occupational Safety and Health [11], the Bureau of Labor Statistics [1,3,12,13], and the Public Health Institute [5]. This data will be used to describe the degree of hazard exposure and priorities for risk identification; develop training approaches for risk reduction and intervention strategies.

## 3. Results

### 3.1. Review of Significant Occupational Health Hazards

Significant occupational health hazards are categorized as five types of hazards outlined by Rogers (2003) [14]: (1) biological/infectious; (2) chemical; (3) enviromechanical; (4) physical; and (5) psychosocial. Biological hazards are infectious agents capable of being transmitted to others via contact with infectious patients or their bodily fluids (e.g., bacteria, viruses, fungi). Chemical hazards refer to any form of chemical “including medications, solutions, gases, vapors, aerosols, and particulate matter that is potentially toxic or irritating to the body system” ([14], p. 148). Enviromechanical hazards are those aspects of the workplace that can cause or potentiate accidents, injuries, strains, or discomfort (such as insufficient equipment or hazardous flooring). Physical hazards are workplace agents that can cause tissue damage by transfer of energy from the agent (e.g., noise, radiation). Psychosocial hazards are those factors that can cause or potentiate stress, strain, or interpersonal problems of the worker. Literature about each of the aforementioned hazards that was reviewed can be found summarized in Table 1, Table 2, Table 3, Table 4 and Table 5.

### 3.2. Biological Hazards

As many NAs are women of childbearing age, human immunodeficiency virus, hepatitis B virus, hepatitis C virus, varicella-zoster virus, herpes simplex virus, human parvovirus B19, cytomegalovirus, rubella, measles, enteroviruses, mumps, and influenza are of particular concern as they can cause problems for pregnant women and their unborn children [15].

NAs who assist patients with activities of daily living (ADLs) may also come into contact with patients’ urine, feces, sweat, and saliva as they assist with toileting, incontinence care, oral care, and bathing in a variety of settings. In a recent study of home care aides in Massachusetts, a total of 3484 home care aide visits were analyzed, and contact with feces occurred in 13% of all visits by agency-hired aides and as much as 24% of visits by client-hired aides [16]. Direct contact with bodily fluids can expose NAs to the common cold, cytomegalovirus, enteric pathogens, herpes simplex virus, measles/mumps, mycobacterium TB (tuberculosis), pertussis (whooping cough), rubella (German measles), scabies/lice, staphylococcus aureus, groups A and B streptococcus, and varicella (chicken pox). NAs who come into contact with needles contaminated with bodily fluids (in addition to exposure to the sharp device itself, discussed in greater depth in enviromechanical hazards below) are also at increased risk for exposure to and infection by bloodborne pathogens.

Influenza has received increasing attention as it is so widespread and can be fatal. A recent study estimated the annual number of occupational exposures to influenza among healthcare workers that result from providing direct and supportive care to influenza patients in acute care, home care, and long-term care settings at 81.8 million. Among the approximately 14 million healthcare workers, this corresponds to an average of 5.8 exposures per worker annually. Occupational exposures were most common in ambulatory care settings (38%), followed by long-term care facilities (30%) and home care settings (21%). The annual number of occupational exposures to influenza is high, but not every occupational exposure will result in infection [17]. An examination of NNAS data found that influenza vaccination rates among NAs are similar to that of the rest of the healthcare worker population (37.1%) [18], but a smaller study examining vaccination rates (*n* = 1042 healthcare workers/135 NAs) found that NAs were significantly less likely than physicians or medical students to get vaccinated but as likely as nurses to get vaccinated. The authors raised concern about the relative patient contact of each of those groups and recommended more focus on vaccination for NAs [19].

### 3.3. Chemical Hazards

Chemical hazards in the workplace for NAs include exposure to antimicrobial/antibiotic drugs, antineoplastic agents (e.g., chemotherapy), antiseptic/disinfectant agents, ethylene oxide (a gas sterilant), formaldehyde, bleaches, rubber products/adhesives, soaps/detergents, and solvents (e.g., acetone).

NAs may become exposed to chemotherapy in the air; contact with work surfaces and clothing, and medical equipment; during spills of liquid chemotherapy in the work environment; and through handling patients’ bodily fluids; urine, feces, and emesis [11]. Chemotherapy is excreted in urine [20]. Antineoplastic drugs, such as chemotherapy, have been found in the urine of pharmacists, pharmacy technicians, and family members caring for patients [21], as well as on home [21] and nursing area work surfaces [22], but documentation of NA exposure outside of a case study [20] is missing. Furthermore, some NAs in the home health setting are administering oral antineoplastic agents, whether it is in their scope of practice and training or not [23], which offers another opportunity for exposure to chemotherapy for NAs who are not trained to handle it.

In a recent Massachusetts study of health care aides that examined what took place in 3484 home care visits, the great majority of the visits included cleaning and disinfecting bathrooms and kitchens (80% visits). Bleach was the most commonly used disinfectant (20–34% of the visits), but ammonia and other strong chemicals were also used [16]. Infection is understood to be a significant concern in home care but some chemicals can also introduce respiratory hazards for the workers handling them [24]. Antimicrobial pesticides are commonly used in healthcare settings for disinfection. Between 2002 and 2007, 401 antimicrobial pesticide work-related illnesses occurred in four states; most cases occurred among janitors/housekeepers and nursing/medical assistants, usually due to splashes or spills, and the eyes were the most common organ/system affected. The agents that caused the illnesses were quarternary ammonium compounds, glutaraldehyde and sodium hypochlorite [25]. Some volatile organic compounds (VOCs) such as benzene, ethylbenzne, toluene, *o*-xylene, and *m*,*p*-xylene are associated with irritant-induced asthma [26]. In a study of different classes of healthcare workers, NAs were found to have the highest personal levels of exposure to total VOCs measured by geometric mean in an evacuated canister [27]. The 14 VOCs tested in that study included the five named above but also included ethanol, and in sub-analyses, NAs had far more exposure to ethanol than other occupations (perhaps because of their use of hand sanitizer).

### 3.4. Enviromechanical Hazards

Enviromechanical hazards in the workplace for NAs include contaminated air, poor ventilation, poor lighting, poor security or proximity of parking, lifting/pushing/pulling of objects, poorly designed or inadequate work area/equipment, contaminated needles, slippery/cluttered floors, splashes/spills/flying particles, and violence/physical assault by both patients and co-workers.

According to the U.S. Department of Labor Bureau of Labor Statistics, in 2015 NAs, laborers and freight, stock, and material movers and heavy tractor-trailer truck drivers incurred the highest number of musculoskeletal disease (MSD) cases [13]. Each of those groups incurred at least 5% of the total private sector MSD cases in 2005 [13]. The recent rate for NAs is on the decline and was 171 cases per 100,000 full-time workers in 2015, down from 191.1 cases in 2014 [13]. In the inpatient setting, NAs are twice as at risk for injury compared to nurses [28], and despite the advent of lifting equipment, the Occupational Health and Safety Network (OHSN) surveillance system for healthcare facilities found the same increased incidence for NAs compared to nurses in 2012–2014 [29]. The OHSN also found that of the patient handling injury reports, 62% included data on the use of lifting equipment and 82% of the injuries occurred when lifting equipment was not used [29].

In a recent survey of randomly sampled NAs registered with their state board of nursing about back injuries while working, Graham and Dougherty [30] reported that 46% of the respondents reported having hurt themselves while lifting, moving or helping a patient and 40% of respondents incurred a back injury. NAs also reported twisted arms, pulled shoulder muscles, needlestick injuries, and other injuries that were directly the result of violence such as pulled hair, bites, scratches, and skin breaks. The majority of respondents were working in nursing homes at the time of the injury (79%). In this survey, NAs were also asked about the hardest aspects of their job and the authors related some of these narratives as proxies for potential and actual barriers to safe patient handling. Among the ones to emerge were too many patients to manage, inadequate staffing, poor communication, lack of teamwork, low pay, and patient transfers. A recent review found that work-related musculoskeletal risks (sprains/strains, low back pain, and wrist, knee and shoulder injuries) increased when nurses and NAs were manually moving or lifting patients, especially when the patients were overweight or obese [31]. A review of workers’ compensation claims in an acute care setting found that NAs had higher overall injury rates than nurses for no-lost work time and lost work time [32]. In a study at two large academic medical centers, similar results were found [33]. The risk of an injury due to lifting was greater among NAs compared to nurses for both non-lost work time and lost work time injuries. Injury rates among NAs were particularly high in rehabilitation and orthopedics units, suggesting that certain worksites may be targeted for intervention efforts [32]. NAs were also found to be more likely to have repeat injuries than their nursing colleagues [34].

Related to this discussion of back injuries and patient lifting is sufficient availability of lift equipment. Several studies have found that assistive equipment reduces the incidence of injuries [35,36,37]. However, two studies have also found no statistically significant relationship between worker injury rates and availability of safety equipment [38,39]. Descriptive data from the NNAS and NNHS found that assistive equipment is widely available and most NAs use it. However, among NAs who reported never using or only sometimes using equipment, many had access to equipment [38], which is important to understand in light of the fact that lifting equipment was often not used in patient handling injury reports [29]. The authors suggest additional research to understand the reasons for non-use among those who report using lifting equipment never or sometimes, but hypothesize that it may be too cumbersome and/or that it may require the help of other unavailable staff. Many NAs report feeling rushed, and it may take time to wait for another staff member to assist or to wait for the equipment to become available [38].

Needle stick injuries are one of the more commonly considered enviromechanical hazards. In a descriptive study which compared workers’ compensation claims in Washington State for needlestick injuries in hospital and non-hospital settings over a 4-year period, it was found that claims in hospitals were largely due to suturing and other surgical procedures (16.7%), administering an injection (12.7%), and drawing blood (10%). For skilled nursing facilities, needlestick injuries were most common during disposal (23.7%) and administering an injection (14.9%). The incidence of needlestick injury claims per 10,000 full-time-equivalent healthcare worker in hospitals was 158.6, in skilled nursing facilities this was 80.8, and in non-hospital settings it was on the rise. The authors argued more attention should be paid to these injuries occurring in non-hospital settings [40].

Administering medications via injection is not within the scope of practice of NAs but a recent study of home care aides employed in a variety of medical and social service systems in Massachusetts reported that client-hired health care aides reported helping a client use a needle or lancet seven times more often than agency-hired health care aides. They also found that fewer client-hired NAs knew how to report sharps injuries. Agency-hired health care aides are trained not to use sharps. However, the authors suggested that because of the close client relationship, it may be difficult for the client-hired health care aide to resist assisting with diabetes management or vitamin injection which is outside of their scope of practice when asked by the client [16].

Slips, trips, and falls are also common for NAs. Of all injuries due to slips, trips, and falls reported in the OHSN surveillance system, 65% had data on fall type; 89% were falls on the same level, 9% were falls down to a lower level (e.g., down stairs, ramps, etc.), and 2% were slips and trips without falling [29].

Data from the NNAS and NNHS were linked to examine the prevalence and contributing factors to injuries caused by assault at the individual and organizational levels. Mandatory overtime, not having enough time to assist residents with activities of daily living, and race/ethnicity (being Hispanic/Latino) were highly associated with reports of assault and human bites. Older NAs had fewer assault injuries, and NAs in nursing homes with Alzheimer’s units had a significantly higher risk of assault/bites after adjustment for other individual-level factors [41]. Age was inversely related to assault in two other studies [42] with older NAs being assaulted less than younger NAs [43]; in a study that included a majority of respondents holding the title home care aide and not NA, no correlations with age were found [44]. Another analysis of the NNAS and NNHS data sets examined all types of injuries that occurred at work and found that of the 1738 of 2886 surveyed NAs that experienced an injury in the past year, 44.6% reported scratches, open wounds, and cuts; 16.2% reported black eyes and bruising; and 11.5% reported human bites. A substantial portion of the injuries were the result of violence [38].

In a separate study of the OSHN database which included 2034 workplace violence injuries over a 2-year period in 112 institutions, NAs had twice the injury rate of nurses for workplace violence injuries. Between 2012 and 2014, workplace violence injury increased for all job classes but nearly doubled for NAs and nurses. Of all workplace violence injury reports, 49% specified the type of assault (among physical, verbal, or destruction of property), and 99% were physical assaults. Descriptions of who caused the assaults were included in 13% of workplace violence injury reports with 95% caused by patients [29]. This is a consistent finding with other literature [41]. In a study that investigated aggressive incidents from patients against NAs in six geriatric care facilities, it was found that there was a 95% underreporting of the incidents. Among the reasons NAs cited for not reporting were the lack of intention to harm the NA, the lack of a serious injury, expecting such incidents to occur, too difficult/time consuming to report, lack of action on complaints by administration, and no requirement of reporting [45]. This finding suggests that estimates of assault in other literature may be low.

A recent survey of nurses and NAs at three long-term care facilities found that 65% of the respondents experienced workplace violence, 41% reported that management showed little or no concern for their safety, and 22% of those who had experienced workplace violence reported that the work environment was not safe to perform their duties. Again, patients, followed by co-workers, were found to be the most common perpetrators of violence [46]. In a study of 138 NAs across six nursing homes, 59% of NAs said they were assaulted once per week and 16% said they were assaulted daily. Fifty-one percent reported assault that resulted in injury and 38% of those injured received medical attention [42]. In a study of nursing home workers responding to three consecutive annual surveys (*n* = 344), 34% of the respondents, the majority of whom were NAs (*n* = 243), reported persistent workplace assault over two years. Taken together with other job groups, among respondents assaulted frequently, two-thirds had moderate to extreme musculoskeletal pain, and more than half had pain that interfered with work and/or sleep. Pain caused by assault may affect NAs’ ability to remain employed [47].

Another study included 282 NAs working across five large urban nursing home and utilized the resident as the unit of analysis to examine individual and organizational factors that contribute to resident to staff aggression. Staff reported that 15.6% of residents directed aggressive behaviors towards them (2.8% physical, 7.5% verbal, 0.5% sexual, and 4.8% both verbal and physical). Aggressive behaviors occurred most commonly in resident rooms (77.2%) and in the morning (84.3%), during the provision of morning care, suggesting that providing help with activities of daily living in the morning increased risk for aggressive behaviors towards staff. Three clinical factors were significantly associated with resident-to-staff aggression: greater disordered behavior, affective disturbance, and need for activities of daily living morning assistance [48]. Myers et al. found similar results regarding contact with residents [49].

### 3.5. Physical Hazards

Physical hazards in the work environment for NAs include electricity/fire, extreme heat or cold exposure, noise, and radiation (or hazardous radioactive waste). For example, in the use of laser therapies, laser radiation absorption can result in thermal damage when the laser radiation raises the temperature of body tissue [50]. This type of exposure while performing laser procedures can result in eye injuries, which are the most common, as well as skin burns and electric shock. Pierce (2011) reported on 37 cases of laser-induced injures to health care workers more broadly (e.g., technicians, laser operators, ancillary medical staff) which occurred while performing medical procedures; 73% caused eye injuries, some of which were permanent. Personal protective eyewear was not always worn and is essential in preventing direct and indirect beam exposure [50]. Also, it has been reported that radiation exposure can occur through exposure to urine and feces from patients treated with Radium-223 and Iodine-131 [51,52] which is a care task of NAs. Other types of physical hazard exposure such noise exposure are well-documented in relation to registered nurses [53] and would likely be the same for NAs; however, this is clearly an area that has been understudied in this population.

### 3.6. Psychosocial Hazards

Psychosocial hazards for NAs include concern about hazardous occupational exposures, concern about violence directed towards them, heavy workload, high levels of responsibility, incivility/disrespect and bullying by coworkers, incivility/disrespect and bullying by supervisors/managers, poor staffing, lack of managerial support, long hours and double shifts, physical demands of the work, sexual harassment, and shiftwork. While some of these hazards might better be classified as organizational factors, many of these (long hours, double shifts, lack of managerial support) tend to create stress and psychosocial issues for workers and are included here.

Not all violence that NAs experience is physical. Verbal violence is a common form of horizontal violence; harmful behavior via attitudes, actions and words directed at workers by their colleagues. Bullying is a similar concept and is described as repeated, health-harming mistreatment of one or more people by one or more perpetrators in the form of verbal abuse, threatening, humiliating or offensive behaviors or actions. Horizontal violence and bullying can negatively affect the work environment [54]. Studies by Secrest et al. and Ejaz et al. report that the work environment may be characterized by hostility, disrespect, and lack of control, as well as racially biased comments [10,55]. Racism and negative interactions were significant predictors of job satisfaction for these workers [10]. In an analysis of NNAS data, it was found that black CNAs were three times more likely to report job strain, compared with white CNAs. Black workers, across job categories, reported less perceived control, earned $2.58 per hour less, and worked 7.1 h on average more than their white counterparts. The authors argue that differential results by race and ethnicity may demonstrate interpersonal and/or institutional racism, and that race/ethnicity must also be considered in the context of occupational stress [56].

Being required to work overtime increased the odds of being injured by nearly 80% in multivariate analysis from the NNAS and NNHS [38]. Twenty-two percent of the NAs who responded to the NNAS reported working mandatory overtime [38]. Furthermore, Khatutsky and colleagues also found that 88% of facilities surveyed in the 2004 NNHS reported that their registered nurses, licensed practical nurses, or NAs worked overtime shifts in the week prior to the survey [38]. NAs working overtime are tired, which may make them more prone to making mistakes. Mandatory overtime may also be related to staffing shortages. Other studies have also shown that evening and night shifts in particular are associated with increased workers’ injuries [35,57], and that both physical assaults and human bites were significantly higher among those NAs who reported mandatory overtime [41].

In a survey of 473 U.S. female NAs working in nursing homes in 2004, working two or more double shifts per month was associated with an increased risk for mental health indicators of depression, anxiety and somatization, working 6–7 days per week was associated with depression and somatization and the depression increased significantly with working increased hours or with working rotating shifts [58]. The Center for Epidemiological Studies-Depression (CES-D) measure is often used to assess depressive symptoms. Scores range from 0 to 60, with higher scores indicating greater depressive symptoms [59]. In a study by Ejaz and colleagues, 26% of direct care workers studied (most of whom are NAs) had CES-D scores of 16 and above, indicating risk for clinical depression [10]. In a study of 395 NAs in 49 nursing homes in two states; a striking 59% had symptoms of clinical depression (CES-D scores of 16 and above) and there was a correlation between their symptoms, age, and workplace emotional strain [60]. Private for-profit ownership, emotional strain, non-seniority based wage increases, and managerial domination have also been found to be predictive of depression [61,62].

More than one-third of all NAs reported not having enough time to help with activities of daily living for nursing home residents. D’Arcy et al. [63] using 2004 NNAS data, found that the odds of an injury were lower among NAs who reported sufficient time to complete resident activities of daily living (35%). Communication and a collaborative work environment may also be impacting the time to help with ADLs [38]. Horwitz and McCall found that evening and night shift hospital workers have significantly higher risk of workplace injury than employees working the day shift, as well as longer periods of local disability (though injury severity may not be substantially different between shifts); fatigue, differences in staffing and task differences in shift may be to blame for the differences [57]. Furthermore, in the Nursing Home Nurse Aide Job Satisfaction Questionnaire (2006), NAs rated feeling part of a team effort as low. The authors reported that NAs often work alone or in pairs and that more communication channels could improve the feeling of teamwork [64]. Data from a study that included in-depth interviews with 338 NAs at 22 skilled nursing facilities showed that it was common for NAs to have unexpected changes in their work schedules. Interestingly, more frequent schedule changes were related to better relationships with staff and residents as well as satisfaction with supervision, indicating that better teamwork may help in managing frequent scheduling changes. In the same study, NAs reported twice as many resident friends as they did other NA friends [65]. Staffing shortages and interruptions by nurses and administrators also contributed to the disruption of routine and feeling of lack of control [55].

In a survey of all nursing staff (RNs, LPNs, NAs) at a nursing home in the Southeastern U.S., stress and burnout were also examined to see if they were correlated and results were significant. NAs reported a moderate level of stress and burnout while RNs reported the highest stress levels of the three groups [66]. This finding is counter to a study done by Peterson and colleagues, who administered the Formal Caregiver Stress Index to 72 participants (2/3 of whom were NAs) before and after a course on Alzheimer’s care. The NAs’ average stress scores consistently hovered around 25 points higher than those of non-NAs they were compared to [2].

In the analysis of 2004 NNAS and NNHS data by Khatutsky et al. [38], NA staffing ratios (in terms of hours per patient day) were not a significant predictor of injury. That said, an earlier examination and discussion of the same datasets by Tak and colleagues [41] suggested that perhaps improving staffing levels would reduce workload demands and allow staff more time to spend with each resident, minimizing rushing care and removing that risk factor for assault. Increased workload and fewer full-time equivalents (FTEs) have been reported as risk factors for assault and injuries among NAs in nursing homes [67,68,69] Insufficient staffing levels may influence NAs to perform their duties in ways that counter their occupational safety training [70].

Federal regulations mandate NAs receive at least 75 h of training and 16 h of clinical training [4]. Despite this fact, studies using NNAS data show that more than one-third of all respondents feel “not at all prepared” or only “somewhat prepared” to work in nursing facilities. In the multivariate analysis done on data from the NNAS and NNHS, NAs who rated their initial training for working in nursing homes as fair or poor had a greater than 30% likelihood of being injured [38]. Furthermore, in the study by Ejaz and colleagues [10], direct care workers reported better continuing education and orientation to the job had higher job satisfaction. Another study of direct care workers (*n* = 105/91 NAs) suggested that training and job satisfaction were the strongest predictors of workplace injury [71].

In a review of worker’s compensation claims made of the Florida workers’ compensation claims database, weekly pay in dollars was analyzed and it was found that 88.2% of NAs received no pay while on leave, and the author proposed that the lack of pay was because most of them return to work within a few days of an injury for a continuous income and the fact that they view their roles as a career more than a job [54].

In a study by Graham, when asked about the hardest part of their job, NAs replied being “looked down on” and having poor relationships with the nurses with whom they worked [30]. One also reported, “The website for the National Board of Labor lists the job of CNA as “unskilled.” Their description is so ignorant and derogatory!” [72]. In her ethnographic study, Jervis points out that because of their frequent contact with excreta NAs are at risk of being viewed as “polluted people” [73]. Khatutsky et al. reported that the odds of being injured decreased for NAs who felt respected and rewarded on the job and for NAs that felt that their organization valued NA work [38].

Director of nursing tenure is related to reduced turnover and improved retention of NAs [74]. In another analysis at one nursing home in Washington State in the late 1990s, “social disarray” in the nursing home was positively associated with injury incidence. Social disarray was measured as the number of NAs hired in the last 30 days plus the number of NAs who quit in the last 30 days [75].

Support from supervisors is consistently associated with decreased intention to change jobs, improved worker safety, and fewer on the job injuries [74,76]. NAs whose supervisors exhibited positive leadership qualities were less likely to report significant workplace injuries or absenteeism related to injury [76]. Likewise, in a recent analysis of NNAS data, negative and significant relationships were found between workplace injuries and NA ratings of supervisor support the odds of being injured once increasing by 3.11 and the odds of being injured more than three times increasing by 2.02 [70]. In a qualitative study that conducted focus groups with NAs and center administrators and directors of nursing, directors failed to recognize some workplace hazards that emerged as concerns of NAs such as caring for patients with infectious diseases, trip hazards and assault [77]. Leaders have been urged to build on the base of what NAs value—job enrichment opportunities, personal growth opportunities, recognition, responsibility, and sense of achievement [78].

In an analysis of the 2004 NNHS data, the annualized turnover rate was found to be 74.5% among NAs. Longer director of nursing tenure, more RN hours per patient day, and more NA hours per patient day showed associations to lower turnover for NAs, LPNs and RNs [74]. NAs are also less likely to think about leaving, think about a job search, or conduct a job search when they are satisfied with the job’s rewards (such as wages and opportunities for advancement) [64]. In a study that utilized the Nursing Home Nurse Aide Job Satisfaction Questionnaire to survey NAs about their satisfaction, the authors concluded that rewards other than monetary compensation are important to NAs, who rated their chances for further advancement as low [64]. Similarly, a survey of randomly-sampled NAs in Iowa, was compared with those of other occupational groups, and NAs scored lower than the other occupations in involvement, co-worker cohesion and supervisor support, suggesting strategies that might minimize turnover. The same survey found that NAs who had left their jobs rated it high on task orientation and excessive managerial control [79].

## 4. Discussion

Working as a NA is hazardous; we have explored and described five areas of workplace hazards for this large workforce. Our review has described the hazards that appear in the literature in each of these five focused areas. We undertook this work to describe the degree of hazard exposure and priorities for risk identification; develop training approaches for risk reduction and intervention strategies.

We found the fewest articles centered on chemical and physical hazards for NAs. Literature about chemical hazards reviewed suggest that two significant areas for exposure are through hazardous drugs themselves as well as the bodily fluids of patients receiving them and exposure to cleaning chemicals and hand sanitizer [16,27]. While we found limited literature about NA exposures to hazardous drugs and bodily fluids, recommendations for safe handling of bodily fluids exist for NAs, especially with regard to antineoplastic drugs [11]. Furthermore, encouraging NAs to wash their hands with soap and water after contact with patients and after removal of gloves would minimize exposure to alcohol via hand sanitizer and would be beneficial in reducing exposure to biological hazards in the workplace. Exploration of cleaning with less toxic chemicals in the healthcare arena can also be explored. Physical hazards discovered in the literature about NAs included exposure to laser, radiation and noise [50,51,52,53]. In the case of both laser and radiation, again recommendations for personal protective equipment (PPE) exist but the extent to which NAs are trained or use PPE is unknown. Reducing noise in the workplace may be an outcome of interventions that seek to change the workplace climate of NAs.

We found slightly more in the literature about biological hazards that NAs face. Bloodborne pathogens and other illnesses transmitted through the bodily fluids of patients can expose NAs to a host of communicable diseases [15]. Use of PPE is expected to minimize those hazards though it may never be completely eliminated. In a recent feasibility study conducted by the authors in which we inquired about barriers to PPE used when handling bodily fluids of patients receiving antineoplastic drugs, time, workload, immediacy of the need of the patient, lack of knowledge about the hazards of exposure and the behaviors of other NAs were influential in making decisions about whether or not to use PPE [80]. Again, workplace factors have a great influence on the protective behaviors (or lack thereof) of NAs. Influenza also emerged as a significant hazard for NAs, and vaccination rates of NAs who have the most direct contact with patients lag behind that of other providers suggesting need for additional intervention here as well [18,19].

Within the domain of enviromechanical hazards, the majority of literature we reviewed focused on needlestick injuries, back injuries and workplace violence. Administering injections is usually not within the scope of practice of NAs but many report administering them, especially in non-healthcare settings where such injuries are on the rise [40]. The majority of literature about back injuries focused on use (or non-use) of lift equipment, but the finding that provision of lift equipment is not enough to predict its use is compelling and again calls us to examine sufficient staffing, teamwork and workload for NAs who are experiencing the majority of these injuries when compared to other HCWS [13,28,29,30,31,32,33,35,36,37,38,39,63]. Finally, the volume of literature on workplace violence experienced by NAs at the hands of patients and colleagues is striking [29,30,38,41,43,44,45,46] and calls us again to examine the culture of workplace incivility that is pervasive in the healthcare arena today.

The vast majority of the literature we found related to the psychosocial hazards experienced by NAs. Psychosocial stress came from a variety of sources including the threat of violence, bullying, poor teamwork, feeling looked down on, lack of leadership support, feeling rushed at work, feeling unprepared for the job tasks at hand, few opportunities for advancement, low wages, and mandatory overtime [2,10,39,41,42,54,56,57,58,60,61,62,63,64,65,66,67,68,70,71,73,74,75,76,77,78,79,81]. In addition to their frequency in the literature, the effects of these stressors is far reaching, in terms of individual health outcomes for the NAs and in terms of sustaining a workplace climate that allows other hazards to happen. These psychosocial stressors may be the most difficult and the most crucial to intervene upon for NAs.

## 5. Strengths and Limitations

While this manuscript selectively reviewed literature related to risks that fell into the five areas of hazard as described by Rogers [14], many risks emerged and are summarized in Table 6. This is the first review to look at nursing assistant hazards in the context of these five domains. We utilized a wide variety of search terms, and a wide range of dates and studies of different types to cast a broad net on hazards of concern for NAs. The fact that we focused our review only on NAs in the U.S. helps to contextualize the findings.

The greatest limitation to this review may be that we used a targeted way of searching for hazards within five common hazards instead of systematically searching all that exists in the literature about hazards for NAs or following guidelines for reviews as in the case of scoping reviews or systematic reviews. As a result, we do not have a pre-established date range for our search, nor did we undertake an examination of rigor of each of the studies. However, since this is an understudied population, having some characterization of the scope of hazards in the literature is informative. Furthermore, we acknowledge that the use of grey matter such as student dissertations and websites in our review, while helpful in broadening the scope of what is reviewed, presents its own challenges in terms of being able to be found and being exhaustive [82]. Additional hazards may exist that have not been documented and do not appear in the literature. This review only examines what is documented in the literature as hazards for NAs within these five domains.

## 6. Recommendations

We recommend that future research should survey NAs about the priorities they regard as most influential to minimize hazards in their workplaces. We recommend focusing interventions on the parts of work that are most stressful for NAs to improve safety across all domains of hazards. Recommendations for practice include efforts to improve influenza vaccination rates among NAs, improving utilization of recommended PPE when coming into contact with hazardous drugs or the bodily fluids of those receiving them, working against the culture of disrespect and incivility in the workplace, and working to ensure that NAs have some stability in scheduling without mandatory overtime and with sufficient staffing and leadership support.

## 7. Conclusions

Nursing assistants face a myriad of biological, chemical, enviromechanical, physical, and psychosocial hazards that are directly related to their work. Some hazards are clearer than others, such as biological/infectious hazards which arise through exposure to patients’ bodily fluids (which is clear for NAs who perform the majority of ADLs in healthcare settings), yet does not make up a large volume of the literature on hazards NAs face at work. We found it most compelling that the greatest volume of literature about hazards NAs face within these five domains was in the psychological domain and was related to stress from work organization issues. When combined with their existing vulnerabilities as low-wage workers with low educational attainment levels and the health disparities that already exist for people with those characteristics, the necessity of intervention to reduce risk, especially that caused by psychological stress, is underscored. It would be helpful to know which hazards are predominant for NAs and which of those risks they find most concerning and intolerable as priority areas for intervention and future research. Occupational health research that explores and intervenes to attenuate these risks for a large and vulnerable group of workers is needed.

## Figures and Tables

**Table 1:** Summary of literature reviewed for occupational health and safety hazards to nursing assistants—biological hazards.

Study	Selected Outcomes	Design	N	Population
Christini et al., 2007 [19]	Significant differences in vaccination rates occur among employee groups and those with the most contact have the least vaccination rates.	Cross-sectional survey	1042 HCWs	HCWs in two tertiary teaching hospitals in Pennsylvania
Groenewold et al., 2012 [18]	Influenza vaccination coverage for NAs working in nursing homes is estimated at 37%.	Cross sectional analysis of a survey	2873 NAs	NAs participating in the 2004 NNAS
Quinn et al., 2016 [24]	Exposure to potentially infectious agents was one of the most frequently occurring hazards for home care aides.	Cross sectional survey	1249 home care aides	Home care aides in Massachusetts

HCW: health care worker; NA: nursing assistant; NNAS: National Nursing Assistant Survey.

**Table 2:** Summary of literature reviewed for occupational health and safety hazards to nursing assistants—chemical hazards.

Study	Selected Outcomes	Design	N	Population
Kusnetz and Condon, 2003 [20]	A patient care assistant developed allergic reactions after exposure to urine of oncology patients who had been treated with antineoplastic drugs.	Case report	1	Patient care assistant on an inpatient oncology unit
CDC, 2010 [25]	From 2002 to 2007, 401 cases of illness from antimicrobial pesticides were reviewed and the most common groups affected were housekeepers and medical/nursing assistants.	Retrospective review of data on antimicrobial pesticide related illness	Review of 401 cases of illness	Records came from California, Louisiana, Michigan and Texas
LeBouf et al., 2014 [24]	Identified and analyzed volatile organic compound exposure profiles of HCWs. Found that NAs had the highest exposures overall and especially to ethanol.	Analysis of personal and mobile area evacuated canisters	14 types of HCWs across five hospitals	Location of hospitals not given (3 VA hospitals and 2 teaching hospitals)
Hittle et al., 2016 [23]	Home health aides are exposed to urine and fecal matter more often than home health nurses and have similar exposure to saliva.	Cross sectional design-interviews	31 home health nurses and 23 home health aides	Kentucky and Ohio
Quinn et al., 2016 [16]	Exposure to cleaning chemicals was one of the most frequently occurring hazards for home care aides.	Cross sectional survey	1249 home care aides	Home care aides in Massachusetts

HCW: health care worker; NA: nursing assistant; VA: Veterans Affairs.

**Table 3:** Summary of literature reviewed for occupational health and safety hazards to nursing assistants—enviromechanical hazards.

Study	Selected Outcomes	Design	N	Population
Evanoff et al., 2003 [36]	Reductions in injury and lost day injury rates were greater on nursing units that reported greater use of lifts.	Pre-post intervention study	412 recordable musculoskeletal injuries, interviews of 190 HCWs	Thirty-six intervention units across four hospitals and five long term care facilities in Missouri
Collins et al., 2004 [35]	Significant reduction in resident handling injury incidence, workers’ compensation costs, lost workday injuries after an intervention that included mechanical lifts, repositioning aids, a zero lift policy and employee training on lift usage.	Pre-post intervention study	1728 nursing staff of various types	Six U.S. nursing homes during a 6-year study period (1995–2000)
Gates et al., 2004 [42]	Assaults against NAs from nursing home residents were common; 59% said they were assaulted at least once a week and 16% said they were assaulted daily. Fifty one percent reported injury from resident assault and 38% needed medical attention for the injury.	Survey	138 NAs	Six nursing homes in a large metropolitan area in the Midwest
Myers et al., 2005 [49]	Injuries were associated with resident lifting and assaults were associated with contact with combative residents.	Mixed design cohort study	92 staff (22 RNsand 70 NAs)	A 122-bed long term care facility in New England
Shah et al., 2005 [40]	Needlestick injuries occurred most often in hospitals and to nurses but needlestick injury claims were on the rise in non-hospital settings.	Descriptive study	3303 accepted state funded HCW needlestick injury claims	Washington state HCWs eligible to file a worker’s compensation claim and those who filed a claim for needlestick injury
Nelson et al., 2006 [37]	A multifaceted lift program resulted in an overall lower injury rate, fewer modified duty days taken per injury and significant cost savings.	Pre-post intervention design	825 nursing staff of various titles including NAs	Twenty-three high risk units in seven facilities in the Southeastern U.S.
Snyder et al., 2007 [45]	CNAs experienced a median of 26 aggressive incidents over the course of the 2-week study and approximately 95% of these incidents were not reported to the facility.	Sequential surveys	26 CNAs	Six long term geriatric care facilities in the Rocky Mountain region
Pompeii et al., 2009 [28]	Injury rates were highest for NAs. Forty percent of injuries due to lifting/transferring patients may have been prevented through the use of mechanical lift equipment, while 32% of other injuries would not have been prevented by the use of lift equipment.	Review of workers’ compensation records	19,487 workers making 861 claims (199 were from NAs)	Workers’ compensation records over 5 years (1997–2003) from Duke University Medical Center
Rodriguez & Acosta, 2009 [32]	NAs had higher overall injury rates than nurses for no-lost work time and lost work time injuries. The risk of an injury due to lifting was greater among aides than nurses for both lost work time and non-lost work time injuries. Injuries among NAs were especially high in rehabilitation and orthopedic units.	Retrospective review of personnel records	1689 NAs and 5082 nurses working in acute care	Duke University and Health system employees
Galinsky et al., 2010 [44]	Nearly 5% of respondents reported a patient assault in the year prior to the survey. Three significant risk factors emerged including having one or more patients with dementia, routinely handling patients and perceiving threats of violence by others in and around patients’ homes.	Survey	677 home healthcare aides and nurses	11 home healthcare agencies serving patients in urban and suburban areas of Arkansas, California, Illinois and Oregon
Tak et al., 2010 [41]	34% of NAs surveyed reported physical injuries from resident aggression in the prior year. Mandatory overtime and not having enough time to assist patients with activities of daily living were strongly associated to experiencing injury from assaults. NAs in nursing homes with Alzheimers care units were also more likely to have those injuries.	Survey	2888 NAs (67% of eligible NAs) who were working at the time of survey and missing no information on demographics, work-related assaults, and other work factors.	Data from the 2004 NNAS linked to data from the 2004 NNHS
Welch, 2010 [34]	Compared to their nurse counterparts, practical nurses and NAs had higher cumulative probabilities of multiple reported repeat occupational injury or illness incidents. Study findings suggested there is a complex interplay between environmental factors (e.g., location) and nursing staff demographics (e.g., level of education).	Longitudinal surveillance survey	All VHA nursing employees (*N* = 25,697) who reported an initial (index) incident that occurred between Fiscal Year (FY) 2002 and FY 2005	The VHA Occupational Health Strategic Healthcare Group’s Master Automated Safety Incident Surveillance and Tracking System (ASISTS) Database (MAD)
Boden et al., 2012 [33]	Aides have higher injury rates per 100 full-time equivalent workers (FTEs) than nurses for both injuries involving days away from work (11.3 vs. 7.2) and those involving no days away (9.9 vs. 5.7). Back injuries were the most common days away (DA) injuries, while sharps injuries were the most common no days away (NDA) injuries.	Database review and statistical analyses	A total of 5991 nurses and 1543 aides contributed 3964 and 1008.5 FTEs respectively, to this study, where one FTE was defined as 2000 h	Data from the integrated administrative databases for nurses and aides in patient care units of two large academic hospitals in Boston from 28 September 2008 to 26 September 2009
Graham & Dougherty, 2012 [30]	46% of respondents reported having hurt themselves while lifting, moving or helping a patient, with 40% having incurred a back injury. Eleven were working in nursing homes when the injury occurred. CNAs also reported poor working relationships with RNS as a factor that influenced their perceptions of work.	Survey	35 CNAs	Systematic random sampling of 200 CNAs from the State Board of Nursing’s public list of NAs
Khatutsky et al., 2012 [38]	Select findings: 88% of facilities surveyed in the 2004 NNHS reported that their registered nurses, licensed practical nurses, or NAs worked overtime shifts in the week prior to the survey; 60.2 percent of all CNAs reported a work-related injury in the year prior to the survey; among injured CNAs, 65.8 percent reported being injured more than once in the past year, 16 percent required a transfer to light duty work, and 24 percent were unable to work because of their injury. NA staffing ratios (in terms of hours per patient day) were not a significant predictor of injury; the odds of being injured decreased for NAs who felt respected and rewarded on the job and for NAs that felt that their organization valued NA work.	Descriptive and multivariate analyses of data combined from two large national surveys	2886 CNAs	2004 data from the NNAS and NNHS
Stanev et al., 2012 [39]	Facilities without a lifting policy had a higher estimated injury rate than facilities without such a policy; however, none of the safety equipment was associated with significant changes in injury rates.	Survey	950 nursing home facilities	A survey of Ohio nursing homes in 2007
Lachs et al., 2013 [48]	Staff reported that 15.6% of residents directed aggressive behaviors toward them (2.8% physical, 7.5% verbal, 0.5% sexual, and 4.8% both verbal and physical). Overall, physical aggression toward staff was reported for 7.6% of residents. Aggressive behaviors occurred most commonly in resident rooms (77.2%) and in the morning (84.3%), typically during the provision of morning care. Three clinical factors were significantly associated with resident-to-staff aggression: greater disordered behavior, affective disturbance, and need for activities of daily living morning assistance.	Prevalent cohort study	Population-based sample of 1552 residents (80% of eligible residents) and 282 CNAs	Five large nursing homes in two regions of New York City
Miranda et al., 2014 [47]	34% of nursing aides reported persistent workplace assault over the 2 years. Among respondents assaulted frequently, two thirds experienced moderate to extreme musculoskeletal pain, and more than 50% had pain interfering with work and/or sleep.	Three consecutive annual surveys	344 nursing home workers	Employees in 12 nursing homes within a single company, located in Maryland and Maine
Arnetz et al., 2015 [43]	NAs reported 14.4% of the cases. Specific causes of violence related to patient behavior were cognitive impairment and demanding to leave. Catalysts related to patient care were the use of needles, patient pain/discomfort and physical transfers of patients. Situational factors included the use/presence of restraints, transitions in the care process intervening to protect patients and/or staff, and redirecting patients.	Qualitative content analysis	214 type II violence incidents	Employees of an American hospital system in the Midwest with seven hospitals and a centralized reporting system
Choi and Brings, 2015 [31]	Evidence suggested that the work-related musculoskeletal risks among nurses and NAs included sprains/strains, low back pain, wrist, knee and shoulder injuries. The findings indicated that the workplace musculoskeletal disease risks increased when nurses and NAs were manually moving or lifting patients, especially when the patients were overweight or obese.	Literature review	22 articles	22 referenced articles out of 350 considered related to overweight or obese patient handling
Gomaa et al., 2015 [29]	NAs were more likely to sustain injuries than workers in other job categories; they had more than twice the injury rate of nurses for patient handling and workplace violence injuries.	Analysis of OSHN database	13,798 slips, trips, and falls; patient handling; and workplace violence injuries; 10,680 (77.4%) were OSHA-recordable injuries	Data on injuries occurring from 1 January 2012 to 30 September 2014, from 112 U.S. health care facilities
Fasanya & Dada, 2016 [46]	65% of the participants had experienced workplace violence (WPV) and 41% believed that management showed little or no concern for their safety. 22% of those who reported that they have experienced WPV believed that the work environment is not safe to perform their duties.	Survey	80 nurses and CNAs	Nurses and CNAs working in long-term medical care facilities in North Carolina

HCW: health care worker; NA: nursing assistant; CNA: certified nursing assistant; RN: registered nurse; VHA: Veteran’s Health Administration; NNAS: National Nursing Assistant Survey; NNHS: National Nursing Home Survey; OSHA: Occupational Safety and Health Administration; OHSN: Occupational Health and Safety Network; ADLs: activities of daily living.

**Table 4:** Summary of literature reviewed for occupational health and safety hazards to nursing assistants—physical hazards.

Study	Selected Outcomes	Design	N	Population
Pierce et al., 2011 [50]	Eye injuries, skin burns, injuries related to the onset of fires, and electric shock have been reported in relation to medical laser use. It is probable that both acute and chronic health effects have been experienced by medical personnel as the result of exposure to laser-generated air contaminants.	Literature review and review of 87 incident reports in the Rockwell Laser Industries (RLI) database	37 cases of laser induced injury among HCWs	Events that were reported in the Rockwell Laser Industries (RLI) database
Watson et al., 2015 [53]	Mean noise level was 71.9 dBA. Mean heart rate was 85.2/min and was significantly associated with noise, unit, within-unit location, nurse sources, and noise activities. The most frequent sources of noise were patients’ rooms, care activities, and staff communications.	Cross sectional pilot study	15 nurses	Three units within Cincinnati Children’s Hospital

HCW—health care worker.

**Table 5:** Summary of literature reviewed for occupational health and safety hazards to nursing assistants—psychosocial hazards.

Study	Selected Outcomes	Design	N	Population
Jervis, 2001 [73]	Given their frequent contact with pollutants, NAs are at risk of being viewed as “polluted people”. This has an impact on their status in the workplace, relationships with others and attitude towards work and themselves as workers.	Ethnographic fieldwork; participant-observation, semi-structured interviews	14 residents and 16 staff members	21 months of ethnographic fieldwork from 1993 to 1995 in an inner-city nursing home in a Midwestern city
Myers et al., 2002 [72]	Overall rates of injuries (55.6 per 100 person years) and assaults (67.3 per 100 person-years) were high. Injuries were associated with resident lifting and assaults were associated with contact with combative residents.	Mixed design cohort study	92 employees; 70 CNAs and 22 RNs	A 122-bed long-term care facility in New England, where most were elderly and all had psychiatric disorders. A total of 12 months of data were collected prospectively and data were collected retrospectively for 4 months.
Peterson et al., 2002 [2]	The CNAs’ average stress scores consistently hovered 25 points higher than non-CNA scores. This represented a consistent stress rating almost twice that of non-CNAs.	Pretest-post-test design with a longitudinal component	72 participants (71% of whom were NAs)	Participants were recruited from attendees at a training session offered to 750 long-term care facilities in a 38 county service region near St. Louis, Missouri
Pennington et al., 2003 [78]	Issues important to CNAs revolved around basic motivational factors, such as job enrichment opportunities, personal growth opportunities, recognition, responsibility, and sense of achievement.	Qualitative pilot study	12 CNAs	CNAs working in six Colorado nursing homes
Collins et al., 2004 [35]	There was a significant reduction in resident handling injury incidence, workers’ compensation costs, and lost workday injuries after a best practice musculoskeletal injury intervention designed to safely lift physically-dependent nursing home residents.	Pre-post intervention trial	1728 nursing staff	Six nursing homes in a six year study period (1995–2000)
Geiger-Brown et al., 2004 [58]	Working two or more double-shifts per month was associated with increased risk for all mental health indicators, and working 6–7 days per week was associated with depression and somatization. There was a trend for increasing odds of adverse mental health with increased numbers of demanding work schedule factors. The odds of depression were increased four-fold when working 50 h/week, more than two weekends/month and more than two double shifts/month.	Cross-sectional survey	473 female nursing assistants	NAs working in 49 unionized nursing homes in West Virginia, Ohio and Kentucky over an 8-month period from 1999 to 2000
Horwitz and McCall, 2004 [57]	Evening and night shift hospital employees were found to be at greater risk for sustaining occupational injuries than day shift workers, with those on the night shift reporting injuries of the greatest severity as measured by disability leave. Staffing levels and task differences between shifts may also affect injury risk.	Secondary data analysis	7717 HCWs including NAs	Oregon workers’ compensation claim data from 1990 to 1997. Oregon hospital employee claim data, hospital employment data from Oregon’s Labor Market Information System and shift proportion estimates derived from the Current Population Survey (CPS) were used to calculate injury rate estimates.
Muntaner et al., 2004 [62]	For-profit ownership, emotional strain, managerial pressure, and lack of seniority pay increases were associated with depression.	Cross-sectional survey	539 NAs	NAs working in 49 unionized nursing homes in West Virginia, Ohio and Kentucky over an 8-month period from 1999 to 2000
Gates et al., 2005 [67]	There were significant relationships between assaults and the following covariates: age, state anger, and the number of residents assigned.	Quasi-experimental study	138 NAs	Three intervention and three comparison nursing homes in one county in Ohio
Kennedy, 2005 [66]	Stress was significantly correlated with burnout. RNs had more stress and burnout than did other nursing staff. CNAs reported a moderate level and LPNs reported the least.	Descriptive correlational study	72 NAs, RNs and LPNs	A 252-bed nursing home in the Southeastern U.S.
Trinkoff et al., 2005 [69]	Total nursing hours per resident day were significantly associated with worker injury rates in nursing homes after adjusting for organizational level factors.	Descriptive correlational study	445 Nursing Homes in Ohio, West Virginia and Maryland	First reports of injury and workers’ compensation data from three states (Ohio, West Virginia, and Maryland) for the year 2000, linked to Medicare’s Online Survey, Certification and Reporting system
Muntaner et al., 2006 [60]	Workplace emotional strain and age were associated with increased odds of depression.	Cross-sectional survey	395 NAs	49 nursing homes in Ohio and West Virginia represented by a single union in the fall of 2000
Noelker et al., 2006 [65]	Personal stressors (family, financial, and health concerns) have the greatest impact on satisfaction with supervision. Positive support in the workplace attenuated the effects of job-related stressors on the outcome.	Three cross-sectional surveys of NAs	338 NAs	22 skilled nursing facilities in northeast Ohio
Castle et al., 2007 [64]	Nurse aides enjoy working with residents and their coworkers but are less satisfied with pay.	Survey	1579 NAs	A random facility sample of approximately 10% (*N* = 240) of nursing homes from five random states (New York, Oregon, Michigan, Colorado, and Florida)
Culp et al., 2008 [79]	When CNA responses were compared with those of other occupational groups, general workers reported higher scores on involvement, coworker cohesion, work pressure, and supervisor support. Those who left their CNA jobs rated their work environment as characteristic of excessive managerial control and task orientation.	Population-based, cross sectional study	584 CNAs	Iowa CNAs from 166 nursing homes
Ejaz et al., 2008 [10]	Background characteristics of direct care workers (DCWs) were less important than personal stressors (e.g., depression), job-related stressors (e.g., continuing education), and social support (e.g., interactions with others) in predicting job satisfaction. Nursing homes compared to the two other types of Long Term Care organizations had lower average DCW job satisfaction rates, as did organizations offering lower minimum hourly rates and those with turnover problems.	Survey	644 DCWs	DCWs in nursing homes, assisted living facilities, and home health agencies in a five-county area of northeast Ohio
Castle et al., 2009 [68]	For-profit facilities were less likely to report high injury rates, facilities with a higher average occupancy and belonging to a chain were more likely to report high injury rates. For the staffing characteristics of interest, facilities with high staffing levels of registered nurses were more likely to report high injury rates, whereas those with high staffing levels of NAs were less likely to report high injury rates. For the quality characteristic of interest, facilities of low quality (as measured by quality-of-care deficiency citations) were more likely to report high injury rates.	Cross-sectional study		Data from the OSHA data initiative for 2004, the Online Survey Certification and Recording system representing 2004, and the 2004 Area Resource File.
Zontek et al., 2009 [71]	Tenure (77% of injuries occurred after 1 year of tenure) was significantly correlated with training, satisfaction, organizational climate, and stress. When tenure was greater than 1 year, job satisfaction was a predictor of injury and when tenure was greater than 3 years, both job satisfaction and training were predictors of injury.	Cross sectional survey	111	DCWs who attended the Mountain Area Health Education Center Nursing Assistant Training Day on 5 October 2005
Donoghue, 2010 [74]	The annualized turnover rate was found to be the highest among CNAs at 74.5%, followed by registered nurses at 56.1%, and licensed practical nurses at 51.0%. Director of nursing tenure, registered nurse hours per patient day, and CNA hours per patient day show the most consistent associations to lower turnover and higher retention.	Secondary data analysis	1174 nursing homes	The 2004 NNHS was used as the primary source of data
Tak et al., 2010 [41]	Thirty-four percent of NAs surveyed reported experiencing physical injuries from residents’ aggression in the previous year. Mandatory overtime and not having enough time to assist residents with their activities of daily living were strongly associated with experiencing injuries from assaults. NAs employed in nursing homes with Alzheimer care units were more likely to experience such injuries, including being bitten by residents.	Multilevel analysis of secondary data	2888 NAs	Data from the 2004 NNAS that were linked to facility information from the 2004 NNHS
Lee et al., 2011 [76]	The transformational leadership (TL) model was positively linked to workplace injury in the level of NAs. Injury-related absenteeism was also associated with the TL style, indicating that TL behaviors may help address workplace absence among NAs.	Cross sectional survey	2882 NAs	Data from the 2004 NNAS
Zhang et al., 2011 [77]	Work organization issues and physical and psychosocial workplace hazards were identified by certified nursing assistants but were not mentioned by managers.	Qualitative study	14 center administrators and directors of nursing; 27 focus groups with a total of 81 NAs	Employees at seven nursing homes in Massachusetts, Rhode Island, and Maine
D’Arcy et al., 2012 [63]	The odds of an injury in the past year were lower among NAswho reported always having a lift available when needed, available facility training to reduce workplace injuries, and sufficient time to complete resident activities of daily living. Quality of initial training to prevent work injuries was not significantly associated with injury status.	Secondary data analysis	2692 NAs	Data from the 2004 NNAS
Hurtado et al., 2012 [56]	Black CNAs were more likely to report job strain, compared with white CNAs. Black workers were also more likely to report low control. Additionally, black workers earned $2.58 less per hour and worked 7.1 more hours per week on average, controlling for potential confounders.	Cross sectional study	237 DCWs	Four nursing homes in Massachusetts during 2006–2007
Mohammed, 2013 [54]	In a review of worker’s compensation claims made of the Florida workers’ compensation claims database, weekly pay in dollars was analyzed and it was found that 88.2% of NAs received no pay while on leave, and the author proposed that the lack of pay was because most of them return to work within a few days of an injury for a continuous income and the fact that they view their roles as a career more than a job.	Review of Florida Workers’ Compensation Claims Data	501 CNA claims	Open claims in Florida Workers’ Compensation Bureau of Data Quality and Collection, 2010
McCaughey et al., 2014 [70]	NAs who experience job-related injuries have lower levels of job satisfaction, increased turnover intentions, and are less likely to recommend their facility as a place to work or seek care services. NA injury rates are related to employee ratings of injury prevention training, supervisor support, and employee engagement.	Cross sectional survey/secondary data analysis	3017 NAs	Data from the 2004 NNAS
Muntaner, 2015 [61]	Using two-level logistic regressions, the authors found that private for-profit ownership and higher managerial domination are predictive of depression among NAs even after adjustment for potential confounders and mediators.	Cross sectional survey	868 NAs	NAs were from 50 nursing homes represented by the same labor union organization in the tri-state region area of Kentucky, Ohio, and West Virginia. Data collection took place from winter 1999 to spring 2001.

HCW: health care worker; DCW: direct care worker; NA: nursing assistant; CNA: certified nursing assistant; RN: registered nurse; LPN: licensed practical nurse; VHA: Veteran’s Health Administration; NNAS: National Nursing Assistant Survey; NNHS: National Nursing Home Survey; OSHA: Occupational Safety and Health Administration.

**Table 6:** Examples of hazards faced by nursing assistants in a focused review of the literature.

**Biological/Infectious Hazards: infectious agents capable of being transmitted to others via contact with infectious patients or their bodily fluids**	
Bloodborne pathogens (HepB, HepC, HIV)	Pertussis, measles, mumps, rubella
Common cold, influenza	*Staph aureus*, group A and B *Streptococcus*
TB (tuberculosis)	Cytomegalovirus, Herpes Simplex Virus
**Chemical hazards: medications, solutions, gases, vapors, aerosols, and particulate matter that are potentially toxic or irritating to the body**	
Antimicrobial, antibiotic drugs	Antineoplastic drugs
Antiseptics, disinfectants, soaps, detergents	Bleach
Volatile organic compounds	
**Enviromechanical hazards: aspects of the workplace that can cause or potentiate accidents, injuries, strains, or discomfort**	
Lifting, bending, rotating	Needlesticks
Slips, trips and falls	Assault
**Physical hazards: Workplace agents that can cause tissue damage by transfer of energy from the agent**	
Radiation exposure	Noise exposure
**Psychosocial Hazards: factors that can cause or potentiate stress, strain, or interpersonal problems of the worker**	
Verbal violence	Stress (caused by: mandatory overtime, long hours, changing schedules, shiftwork; feeling rushed, unprepared, lack of managerial support)
